# Synthesis of 2,3-dihydronaphtho[2,3-*d*][1,3]thiazole-4,9-diones and 2,3-dihydroanthra[2,3-*d*][1,3]thiazole-4,11-diones and novel ring contraction and fusion reaction of 3*H*-spiro[1,3-thiazole-2,1'-cyclohexanes] into 2,3,4,5-tetrahydro-1*H*-carbazole-6,11-diones

**DOI:** 10.3762/bjoc.9.62

**Published:** 2013-03-19

**Authors:** Lidia S Konstantinova, Kirill A Lysov, Ljudmila I Souvorova, Oleg A Rakitin

**Affiliations:** 1 N. D. Zelinsky Institute of Organic Chemistry, Russian Academy of Sciences, Leninsky Prospect, 47, 119991 Moscow, Russia

**Keywords:** anthracene-1,4-diones, 1*H*-carbazole-6,11-diones, fused thiazoles, fusion reaction, heterocycles, naphthoquinones, ring contraction, sulfur–nitrogen

## Abstract

Treatment of *N*-substituted 2-(methylamino)naphthoquinones **3** and -anthracene-1,4-diones **4** with S_2_Cl_2_ and DABCO in chlorobenzene gave the corresponding 2,3-dihydronaphtho[2,3-*d*][1,3]thiazole-4,9-diones **1** and 2,3-dihydroanthra[2,3-*d*][1,3]thiazole-4,11-diones **2** by triethylamine addition, in high to moderate yields. The DABCO replacement for *N*-ethyldiisopropylamine in the reaction of anthracene-1,4-diones **4** led unexpectedly to the corresponding 2-thioxo-2,3-dihydroanthra[2,3-*d*][1,3]thiazole-4,11-diones **10**. The reaction of 3*H*-spiro[1,3-thiazole-2,1'-cyclohexanes] **1d**, **2d** with Et_3_N in chlorobenzene under reflux yielded 2,3,4,5-tetrahydro-1*H*-carbazole-6,11-diones **15**, **16**, i.e., ring contraction and fusion products. A plausible mechanism was proposed for the formation of the products.

## Introduction

The 1,4-naphthoquinone structure is common for various natural products. It is associated with numerous biological activities, such as enzyme-inhibitory, antifungal, antibacterial, anticancer, antiproliferative, antiplatelet, anti-inflammatory, antiallergic, and antimalarial ones. Benzoquinones fused with heterocycles containing nitrogen in position 2 are the most promising ones for furthering clinical applications [1]. Meanwhile, in terms of further structural and chemical system modifications, the introduction of other heteroatoms (e.g., sulfur) through the heterocyclic ring incorporation to the quinone system has been one of the most interesting modifications. In fact, 2-alkylthio-1,4-naphthoquinones were found to show cell-growth inhibitory properties [2], and dihydrothienonaphthoquinones appeared to be potent antitumour compounds [3].

In searching for agents with better pharmacological properties, wider activity range, and low side effects it seemed quite promising to incorporate two heteroatoms into the heterocycle attached to the naphthoquinone (e.g., thiazole) core. Despite continuous interest in 1,4-naphthoquinones fused with heterocycles, only a limited number of thiazolonaphthoquinones have been known so far, no general approach to their synthesis has been proposed, and yields are often low. 3-Methyl-2-thioxo-2,3-dihydronaphtho[2,3-*d*][1,3]thiazole-4,9-dione has been shown to possess a fungicidal activity to Pyricularia Oryzae [4–5]. A family of thiazolonaphthoquinones fused on a chain with nitrogen heterocycles (pyrrole [6–7], and triazoles [8]) were found to possess antifungal and antitumor activity toward a number of species causing fungal diseases and toward Walker 256 carcinoma cell lines. The compounds with the thiazoloanthroquinone structure have not been reported in the literature so far. We were interested in developing a general method for the synthesis of naphtho- and anthraquinones from readily available 2-aminonaphtho- and anthroquinones as well as in the study of their chemical properties and side reactions.

**Scheme 1:**
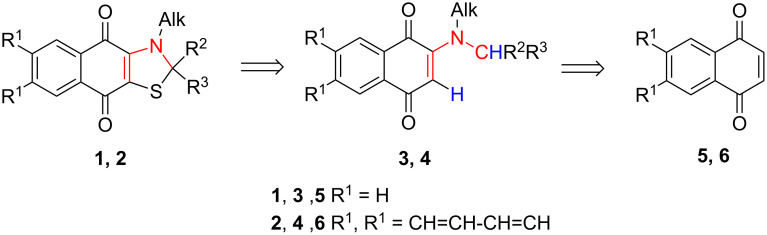
Retrosynthetic analysis of 2,3-dihydronaphtho[2,3-*d*][1,3]thiazole-4,9-diones and 2,3-dihydroanthra[2,3-*d*][1,3]thiazole-4,11-diones.

A retrosynthetic analysis of naphtho- and anthraquinones **1** and **2** led us to a conclusion that the most reliable route would be a reaction of sulfur monochloride with dialkylamino derivatives **3** and **4** (Scheme 1). According to the classification of the synthesis of sulfur-containing heterocycles from organic substrates and S_2_Cl_2_, thiazoles **1** and **2** should be generated from the four-member group shown in red [9]. In turn, dialkylamino derivatives **3** and **4** with two C–H groups activated for the electrophilic attack, one in the α-position to the carbonyl group [10–11] and another located near the nitrogen atom [12], could be prepared from the corresponding naphtho- **5** or anthraquinones **6**.

In this paper we report a study of a reaction between dialkylaminonaphtho- and anthraquinones and sulfur monochloride in the presence of tertiary amines, a selective synthesis of fused thiazoles, and some of their chemical transformations.

## Results and Discussion

**Scheme 2:**
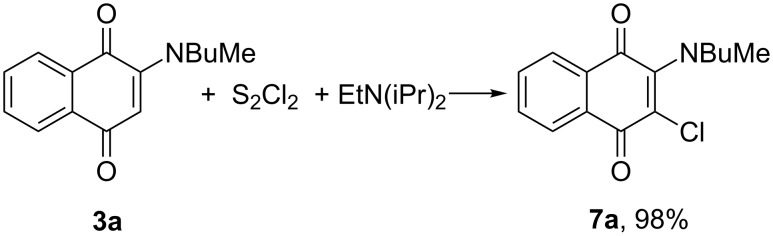
Reaction of 2-[butyl(methyl)amino]naphthoquinone **3a** with S_2_Cl_2_ and Hünig’s base.

We examined in detail the reaction of 2-[butyl(methyl)amino]naphthoquinone **3a** with sulfur monochloride and tertiary amines [*N*-ethyldiisopropylamine (Hünig’s base) and 1,4-diazabicyclooctane (DABCO)]. Treatment of naphthoquinone **3a** with S_2_Cl_2_ (9 equiv) and Hünig’s base (5 equiv) in THF at 0 °C for 72 h with subsequent heating under reflux for 2 h (these reaction conditions had been used in the synthesis of 3,8-dichloroindeno-2*H*-[2,1-*b*]thiophen-2-one from indenylacetic acid [13]) gave with a high yield (81%) only the chlorinated product **7a**. No sulfurated heterocycles were detected in the reaction mixture. The best (practically quantitative) yield of chloride **7a** was achieved by using a 15-fold S_2_Cl_2_ excess (Scheme 2). Reactions of naphthoquinone **3a** with S_2_Cl_2_ and Hünig’s base in other solvents, such as chlorobenzene or acetonitrile, after 1 h under reflux led to the same chlorinated product **7a** albeit in lower yields (70% and 65%, respectively). Sulfur monochloride is known as a powerful chlorinating agent for chlorination of aromatic and heteroaromatic compounds [14–15].

In an attempt to increase the sulfurating ability of S_2_Cl_2_ the complex **8** obtained from S_2_Cl_2_ and 2 equiv of DABCO [16] was used. We have recently shown that this complex can convert *N*-substituted 2-methyl-1*H*-indoles to [1,2]dithiolo[4,3-*b*]indole-3(4*H*)-thiones [15].

**Scheme 3:**
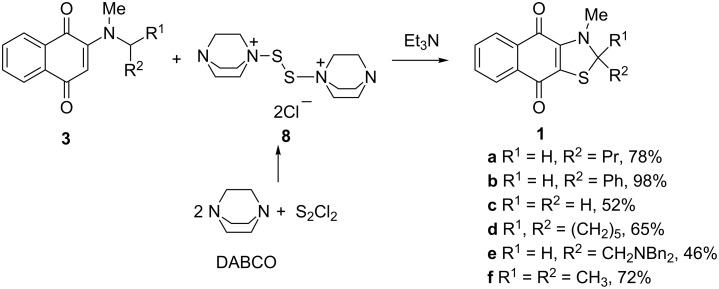
Synthesis of 2,3-dihydronaphtho[2,3-*d*][1,3]thiazole-4,9-diones **1**.

A reaction of naphthoquinone **3a** with a fivefold excess of complex **8** in chlorobenzene for 0.5 h at −20 °C with subsequent treatment with Et_3_N and heating at 100 °C for another 0.5 h, led to a blue solid, mp 71–74 °C. Microanalysis and mass spectrometry data allowed the establishment of its molecular formula as C_15_H_15_NO_2_S. The ^1^H NMR spectrum showed the presence of four aromatic protons (7.05–7.58 ppm), two methyl groups (0.98, 3.40 ppm), two methylene groups (1.48, 1.87 ppm) and one methyne group (5.14 ppm) linked to the methylene group (*J* = 5.5 Hz). The remaining *N*-methyl group (3.40 ppm), loss of one cyclohexandione C–H proton, and butyl CH_2_ fragment conversion to the methyne group undoubtedly supported a novel thiazole structure, **1a** (Scheme 3). The ^13^C NMR spectrum confirmed these observations.

Further on, we extended this reaction to other naphthoquinones **3**. Fused thiazoles **1** were obtained with yields from moderate to high. NMR spectroscopy of thiazoles **1** showed that in all the cases either the primary, secondary or tertiary carbon atom attached to the nitrogen atom was included in the thiazole cycle whereas the *N*-methyl group remained intact, except for dimethylamino derivative **3c** where the *N*-methyl group reacted exactly under the same conditions as with other naphthoquinones **3**, giving 3-methyl-2,3-dihydronaphtho[2,3-*d*][1,3]thiazole-4,9-dione (**1c**) in a moderate yield.

**Scheme 4:**
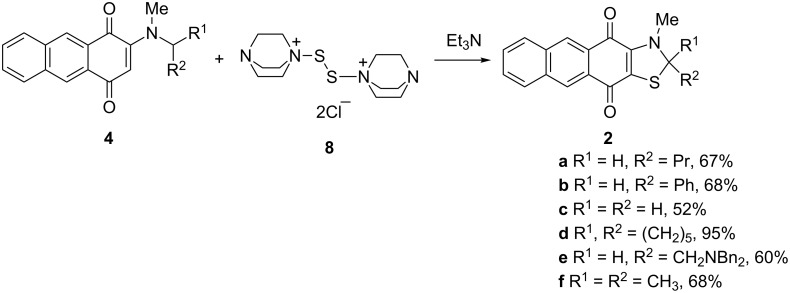
Synthesis of 2,3-dihydroanthra[2,3-*d*][1,3]thiazole-4,11-diones **2**.

The above conditions were further applied to anthraquinones **4** structurally similar to naphthoquinones **3**. Anthraquinonothiazoles **2** were isolated after treatment of dialkylamino derivatives **4** with complex **8** and triethylamine in chlorobenzene (Scheme 4).

**Scheme 5:**
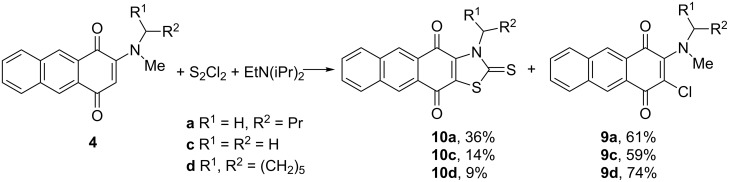
Reaction of *N*-substituted 2-(methylamino)anthracene-1,4-diones **4** with S_2_Cl_2_ and Hünig’s base.

In order to compare the reactivity of anthraquinones **4** and naphthoquinones **3** we studied a reaction of **4** with a mixture of S_2_Cl_2_ and Hünig’s base in THF. After treatment of anthraquinone **4a** with S_2_Cl_2_ (9 equiv) and Hünig’s base (5 equiv) in THF at 0 °C for 72 h with subsequent 2 h heating under reflux we isolated, along with the chlorinated product **9a**, orange crystals **10a**, mp 183–185 °C. Microanalysis and mass-spectrometry data allowed the establishment of its molecular formula as C_19_H_15_NO_2_S_2_, suggesting the presence of two sulfur atoms inserted into the final product instead of four hydrogen atoms. The ^1^H NMR spectrum showed that both the anthraquinone ring and butyl group remained intact whereas three protons of the methyl group and one of the quinone ring disappeared. These observations are in agreement with the thiazole-2-thione structure **10a** (Scheme 5). The ^13^C NMR spectrum confirmed this structure by a characteristic C=S signal at 189.8 ppm. The reaction was then extended to other anthraquinone derivatives **4**. Fused anthraquinonothiazole-2-thiones **10** were isolated in yields ranging from moderate to low together with chlorinated products **9** (with higher yields in most cases).

**Scheme 6:**
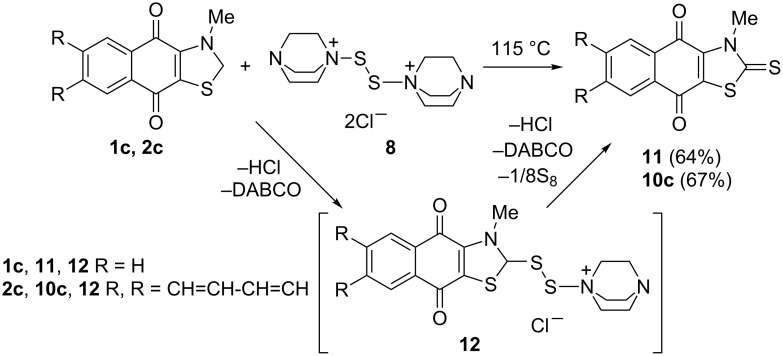
Synthesis of thiazole-2-thiones **10c** and **11** from quinonothiazoles **1c** and **2c**.

To confirm the structure of anthraquinonothiazoles **10** we treated thiazole **2c** with complex **8** in chlorobenzene at 115 °C for 3 h. Thione **10c** identical to the sample prepared from anthraquinone **4** was isolated in moderate yield (67%). Naphthoquinonothiazole **1c** reacted with complex **8** by the same pathway yielding corresponding thiazole-2-thione **11** (Scheme 6).

To the best of our knowledge, there exists only a single example of the thiazole conversion to thiazole-2-thione, i.e., heating of 3-methyl-2,3-dihydro-1,3-benzothiazole with elemental sulfur at 200 °C for 0.5 h giving 3-methyl-1,3-benzothiazole-2(3*H*)-thione [17]. We described a similar conversion of the methylene group to thioketone by the action of sulfur monochloride in the presence of DABCO [18–19]. A plausible mechanism includes the addition of complex **8** to the activated methylene group with formation of S-S-DABCO derivative **12** followed by elimination of a sulfur atom and an HCl molecule [19].

**Scheme 7:**
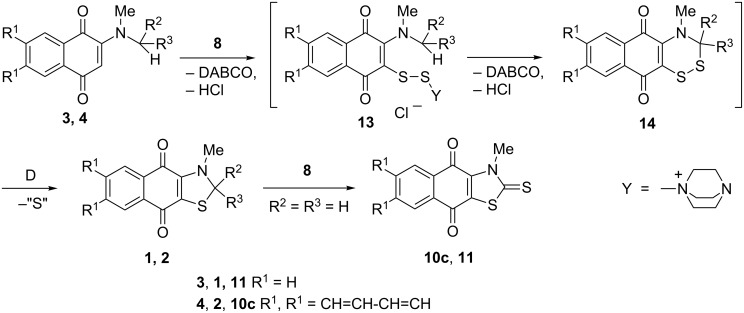
A plausible mechanism for the formation of naphtho- and anthraquinonothiazoles.

We believe that naphtho- (**1**) and anthraquinonothiazoles (**2**) are generated by the same route. The most plausible mechanism is shown in Scheme 7.

The key step is assumed to be the insertion of two sulfur atoms of complex **8** between two activated CH groups, the first one being adjacent to the carbonyl group of the quinone ring and the second to the methyl or methyne group attached to the nitrogen atom with the formation of the six-membered dithiazine ring in **14**, similarly to that proposed for 1,2-dithioles prepared from isopropylamines [14]. Ultimate sulfur extrusion (cf. reference [13]) would then give stable and planar products **1** and **2**.

However, the S_2_Cl_2_ and Hünig’s base combination in THF is preferable in the case of the cyclization of anthraquinone derivatives **4** of the thiazole ring using the *N*-methyl group. It is possible that sulfur in intermediate **13**, which contains the bulkier Y substituent EtN(iPr)_2_^+^ compared to DABCO, attacks the less bulky methyl group giving thiazoles, which are subsequently transformed to thiazole-2-thiones **10** by the further action of S_2_Cl_2_ and Hünig’s base.

**Scheme 8:**
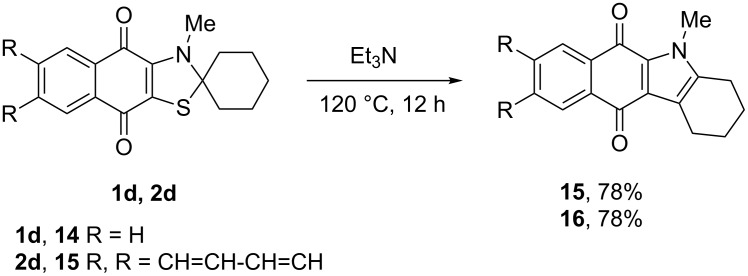
Synthesis of 5-methyl-2,3,4,5-tetrahydro-1*H*-benzo[*b*]carbazole-6,11-dione (**15**) and 5**-**methyl-2,3,4,5-tetrahydro-1*H*-naphtho[2,3-*b*]carbazole-6,13-dione (**16**).

Further study of the reaction between 2-[cyclohexyl(methyl)amino]naphthoquinone **1d** and complex **8** in chlorobenzene allowed us to identify the stable byproduct **15** whose quantity increased by prolonged heating of the reaction mixture, whereas the quantity of thiazole **1d** decreased. This was assumed to be a thermal conversion of **1d** to **15**. Base (e.g., triethylamine) was found to catalyze this reaction and compound **15** was isolated by the heating of **1d** in chlorobenzene at 120 °C for 12 h in 78% yield (Scheme 8). According to mass spectrometry and elemental analysis, this is formally a product of H_2_S elimination, which was confirmed by isolation of triethylammonium hydrogen sulfide in practically quantitative yield from the reaction mixture. The ^1^H and ^13^C spectra showed that the unchanged naphthoquinone cycle and *N*-methyl group are unchanged, while one of the α-methylene groups in the cyclohexane ring was transformed to the tertiary sp^2^ carbon atom. All data are in agreement with the structure of 5-methyl-2,3,4,5-tetrahydro-1*H*-benzo[*b*]carbazole-6,11-dione (**15**) [20].

**Scheme 9:**
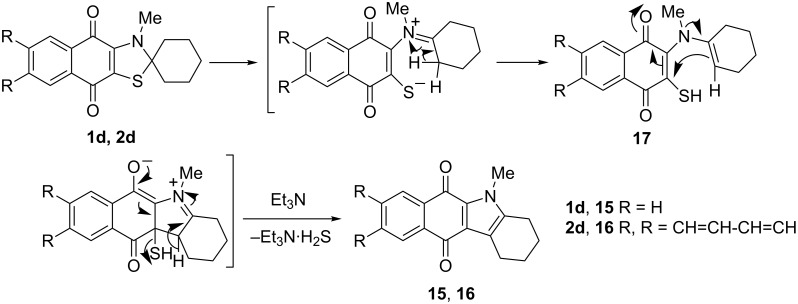
A plausible mechanism for the conversion of spiro compounds **1d** or **2d** into carbazolediones **15** and **16**.

Anthraquinonothiazole **2d** reacted with triethylamine in the same way as naphthoquinone **1d** giving fused pyrrole **16** with the same yield. The most plausible pathway for this reaction is given in Scheme 9.

The key steps are assumed to be the thiazole ring opening with the formation of thiol **17** followed by the dihydropyrrole ring closure under the impact of both quinone and amine groups and the generation of the aromatic pyrrole cycle with hydrogen sulfide extrusion by the action of base (triethylamine). Although, to the best of our knowledge, the transformation of 3*H*-spiro(thiazol-2,1'-cyclohexanes) to tetrahydroindoles has been so far unknown, similar dehydration of spirooxazolocyclohexane to tetrahydroindoles by treatment with base has been previously described [21–22].

## Conclusion

The reaction of *N*-substituted 2-(methylamino)naphthoquinones and -anthracene-1,4-diones with S_2_Cl_2_ provides a short and convenient route to 2,3-dihydronaphtho[2,3-*d*][1,3]thiazole-4,9-diones and 2,3-dihydroanthra[2,3-*d*][1,3]thiazole-4,11-diones, which are of special interest as biologically active compounds. A striking difference in the influence of 1,4-diazabicyclooctane and *N*-ethyldiisopropylamine in the reaction of 2-(methylamino)anthracene-1,4-diones with sulfur monochloride was discovered and explained. 3*H*-Spiro(thiazol-2,1'-cyclohexanes) underwent a new ring contraction and fusion reaction resulting in the formation of tetrahydroindoles.

## Experimental

Melting points were determined on a Kofler hot-stage apparatus and are uncorrected. IR spectra were recorded on a Specord M-80 instrument in KBr pellets. ^1^H NMR were recorded on a Bruker WM 250 spectrometer (250 MHz), and ^13^C NMR spectra were recorded on a Bruker AM 300 (75.5 MHz) in CDCl_3_ solution. *J*-values are given in hertz (Hz). Mass spectra were recorded on a Finnigan MAT INCOS 50 instrument by using electron impact ionization. High-resolution mass spectra (HRMS) were measured on a Bruker micrOTOF II instrument by using electrospray ionization (ESI) [23]. The measurements were done in a positive-ion mode (interface capillary voltage – 4500 V) or in a negative-ion mode (3200 V); mass range from m/z 50 to 3000 Da; external or internal calibration was done with Electrospray Calibrant Solution (Fluka). A syringe injection was used for solutions in acetonitrile, methanol or water (flow rate 3 μL/min). Nitrogen was applied as dry gas; the interface temperature was set at 180 °C. 2-(Dialkylamino)naphthoquinones **3** were prepared as described [24]. 2-(Dialkylamino)anthracene-1,4-diones **4** were prepared from anthracene-1,4-dione by a similar procedure [25].

**General procedure for the synthesis of 3-methyl-2,3-dihydronaphtho[2,3-*d*][1,3]thiazole-4,9-diones 1 and 3-methyl-2,3-dihydroanthra[2,3-*d*][1,3]thiazole-4,11-diones 2:** Sulfur monochloride (0.40 mL, 5.00 mmol) was added dropwise to a stirred solution of 1,4-diazabicyclooctane (1.12 g, 10.00 mmol) in chlorobenzene (80 mL) at −30 °C. The reaction mixture was stirred for 1 h at room temperature, and a solution of the appropriate 2-(dialkylamino)naphthoquinone **3** or 2-(dialkylamino)anthracene-1,4-dione **4** (1.00 mmol) in chlorobenzene (40 mL) was added at −30 °C. The reaction mixture was stirred for 0.5 h at −20 °C, and triethylamine (1.4 mL, 10 mmol) was added at this temperature. The reaction mixture was stirred for 0.5 h at 100 °C and filtered, the solvent was evaporated under reduced pressure, and the residue was separated by column chromatography (Silica gel Merck 60 pretreated with triethylamine, hexane to hexane/CH_2_Cl_2_ mixtures).

**General procedure for the reaction of 2-(dialkylamino)anthracene-1,4-diones 4, sulfur monochloride and Hünig’s base:** Sulfur monochloride (0.72 mL, 9.00 mmol) was added dropwise to a stirred solution of the appropriate 2-(dialkylamino)anthracene-1,4-dione **4** (1.00 mmol) and *N*-ethyldiisopropylamine (0.86 mL, 5.00 mmol) in tetrahydrofurane (65 mL) under argon at −30 °C. The reaction mixture was stirred for 1 h at room temperature and then heated under reflux for 2 h and filtered, the solvent was evaporated under reduced pressure, and the residue was separated by column chromatography (Silica gel Merck 60, hexane to hexane/CH_2_Cl_2_ mixtures).

**General procedure for the synthesis of 3-methyl-2-thioxo-2,3-dihydronaphtho[2,3-*d*][1,3]thiazole-4,9-dione (11) and 3-methyl-2-thioxo-2,3-dihydroanthra[2,3-*d*][1,3]thiazole-4,11-dione (10с) from 3-methyl-2,3-dihydronaphtho[2,3-*d*][1,3]thiazole-4,9-dione 1c and 3-methyl-2,3-dihydroanthra[2,3-*d*][1,3]thiazole-4,11-dione (2c):** Sulfur monochloride (0.40 mL, 5.00 mmol) was added dropwise to a stirred solution of 1,4-diazabicyclooctane (1.12 g, 10.00 mmol) in chlorobenzene (80 mL) at −30 °C. The reaction mixture was stirred for 1 h at room temperature, and a solution of the appropriate quinonothiazole **1c** or **2c** (1.00 mmol) in chlorobenzene (40 mL) was added at −30 °C. The reaction mixture was stirred for 0.5 h at −20 °C, and triethylamine (1.4 mL, 10 mmol) was added at this temperature. The reaction mixture was stirred for 0.5 h at 115 °C and filtered, the solvent was evaporated under reduced pressure, and the residue was separated by column chromatography (Silica gel Merck 60 pretreated with triethylamine, hexane to hexane/CH_2_Cl_2_ mixtures).

**General procedure for the synthesis of 5-methyl-2,3,4,5-tetrahydro-1*H*-benzo[*b*]carbazole-6,11-dione (15) and 5-methyl-2,3,4,5-tetrahydro-1*H*-naphtho[2,3-*b*]carbazole-6,13-dione (16):** A solution of spiro compounds **1d** or **2d** (1 mmol) and triethylamine (0.17 mL, 1.20 mmol) in chlorobenzene (20 mL) was heated under reflux for 12 h. The solvent was evaporated under reduced pressure, and the residue was crystallized from hexane/CH_2_Cl_2_ mixture.

## Supporting Information

File 1 Spectroscopic and analytical data.
