# Supplementary material for: Synthesis of 2,3-dihydronaphtho[2,3-d][1,3]thiazole-4,9-diones and 2,3-dihydroanthra[2,3-d][1,3]thiazole-4,11-diones and novel ring contraction and fusion reaction of 3H-spiro[1,3-thiazole-2,1'-cyclohexanes] into 2,3,4,5-tetrahydro-1H-carbazole-6,11-diones
Source: Beilstein J Org Chem. 2013 Mar 19;9:577–84. doi: 10.3762/bjoc.9.62 (PMC3628778; doi:10.3762/bjoc.9.62)
Supplement: File 1 — Spectroscopic and analytical data. [file Beilstein_J_Org_Chem-09-577-s001.pdf]

Supporting Information

for

Synthesis of 2,3-dihydronaphtho[2,3-*d*][1,3]thiazole-4,9-diones and 2,3-dihydroanthra[2,3-*d*][1,3]thiazole-4,11-diones and novel ring contraction and fusion reaction of 3*H*-spiro[1,3-thiazole-2,1'-cyclohexanes] into 2,3,4,5-tetrahydro-1*H*-carbazole-6,11-diones

Lidia S. Konstantinova, Kirill A. Lysov, Ljudmila I. Souvorova and Oleg A. Rakitin*

Address: N. D. Zelinsky Institute of Organic Chemistry, Russian Academy of Sciences, Leninsky Prospect, 47, 119991 Moscow, Russia.

E-mail:

Oleg A. Rakitin*- orakitin@ioc.ac.ru

* Corresponding author

Spectroscopic and analytical data

2-[Butyl(methyl)amino]naphthoquinone (3a). Yield 62%. Orange crystalline solid, mp 85–88 °C. Anal. calc. for $C_{15}H_{17}NO_2$ (243.13): C, 74.05, H, 7.04, N, 5.76. Found C, 74.52, H, 6.88, N, 5.89. ^{1}H NMR ($CDCl_3$) δ: 0.95 (3H, t, CH_3 *J* 7.3), 1.29-1.40 (2H, m, CH_2), 1.61-1.71 (2H, m, CH_2), 3.09 (3H, s, CH_3), 3.57 (2H, t, CH_2 *J* 7.3), 5.82 (1H, s, CH), 7.55-7.61 (1H, m, CH), 7.63-7.68 (1H, m, CH), 7.94 (1H, d, CH *J* 7.3), 8.54 (1H, d, CH *J* 7.3). ^{13}C NMR ($CDCl_3$) δ: 13.9 and 40.7 (two CH_3), 20.1, 30.0 and 54.4 (three CH_2), 106.5, 125.3, 126.5, 131.9 and 133.8 (five CH),

132.7 and 132.9 (two sp^2 tertiary C), 152.1 (C-N), 182.8 and 183.7 (two C=O). MS (EI, 70 eV): *m/z* (%)=243 (M^+, 60), 200 (100), 186 (21), 146 (38), 69 (62), 57 (23), 43 (51). HRESIMS: found *m/z* 244.1332; calc. for $C_{15}H_{18}NO_2$ $[M+H]^+$ 244.1338. IR (KBr): ν_{max}=2958 and 2872 (C-H), 1675 (C=O) cm^{-1}.

2-[[2-(Dibenzylamino)ethyl](methyl)amino]naphthoquinone (3e). Yield 64%. Orange crystalline solid, mp 104–107 °C. Anal. calc. for $C_{27}H_{26}N_2O_2$ (410.20): C, 79.00, H, 6.38, N, 6.82. Found C, 78.73, H, 6.21, N, 6.60. ^{1}H NMR ($CDCl_3$) δ: 2.69 (2H, t, CH_2 *J* 5.9), 2.79 (3H, s, CH_3), 3.60 (4H, s, $2CH_2$), 3.87 (2H, t, CH_2 *J* 5.9), 5.70 (1H, s, CH), 7.16-7.29 (10H, m, 10CH), 7.53-7.58 (1H, m, CH), 7.66-7.70 (1H, m, CH), 7.83 (H, d, CH *J* 7.3), 8.05 (1H, d, CH *J* 7.3). ^{13}C NMR ($CDCl_3$) δ: 41.0 (one CH_3), 51.6, 52.1 and 59.0 (three CH_2), 107.3, 125.3, 126.5, 127.1, 128.3, 128.9, 131.9, and 133.7 (8 CH), 132.8, 132.9 and 138.9 (three sp^2 tertiary C), 152.3 (C-N), 182.8 and 183.6 (two C=O). MS (EI, 70 eV): m/z (%)=410 (M, 2), 210 (100), 181 (12), 122 (15), 106 (40), 91 (58), 55 (23), 43 (15). IR (KBr): ν_{max}=3024 and 2930 (C-H), 1673 (C=O) cm^{-1}.

2-[Isopropyl(methyl)amino]naphthoquinone (3f). Yield 87%. Orange crystalline solid, mp 58–60 °C. Anal. calc. for $C_{14}H_{15}NO_2$ (229.11): C, 73.34, H, 6.59, N, 6.11. Found C, 73.52, H, 6.73, N, 6.01. ^{1}H NMR ($CDCl_3$) δ: 1.26 (6H, d, $2CH_3$ *J* 6.6), 2.88 (3H, s, CH_3), 4.52-4.61 (1H, m, CH), 5.89 (1H, s, CH), 7.59-7.63 (1H, m, CH), 7.66-7.71 (1H, m, CH), 7.96 (1H, d, CH *J* 7.3), 8.03 (1H, d, CH *J* 7.3). ^{13}C NMR ($CDCl_3$) δ: 20.1 and 32.0 (two CH_3), 51.6, 107.8, 125.4, 126.6, 132.1 and 133.8 (6 CH), 132.8 and 133.1 (two sp^2 tertiary C), 153.7 (C-N), 183.1 and 184.0 (two C=O). MS (EI, 70 eV): *m/z* (%)=229 (M^+, 87), 214 (92), 186 (63), 146 (57), 115 (51), 101 (56), 89 (68), 82 (89), 72 (100). IR (KBr): ν_{max}=2964 and 2873 (C-H), 1673 (C=O) cm^{-1}.

2-[Butyl(methyl)amino]anthracene-1,4-dione (4a). Yield 46%. Orange crystalline solid, mp 80–82 °C. Anal. calc. for $C_{19}H_{19}NO_2$ (293.36): C, 77.79, H, 6.53, N, 4.77. Found C, 77.52, H, 6.48, N, 4.89. ^{1}H NMR ($CDCl_3$) δ: 0.99 (3H, t, CH_3 *J* 7.3), 1.34-1.45 (2H, m, CH_2), 1.66-1.74 (2H, m, CH_2), 3.15 (3H, s, CH_3), 3.65 (2H, t, CH_2 *J* 7.3), 5.98 (1H, s, CH), 7.59-7.66 (2H, m, 2CH), 7.59-8.03 (2H, m, 2CH), 8.51 (1H, s, CH), 8.54 (1H, s, CH). ^{13}C NMR ($CDCl_3$) δ: 14.0 and 40.8 (two CH_3), 20.2, 30.2 and 54.6 (three CH_2), 108.6, 126.7, 128.5, 129.0, 129.3, 129.8 and 129.9 (7 CH), 129.5, 130.0, 134.3 and 135.4 (four sp^2 tertiary C), 153.2 (C-N), 182.0 and 183.3 (two C=O). MS (EI, 70 eV): *m/z* (%)=293 (M^+, 43), 250 (100), 101 (25), 55 (21). HRESIMS: found *m/z* 294.1489; calc. for $C_{19}H_{19}NO_2$ $[M+H]^+$ 294.1494. IR (KBr): ν_{max}=2955 and 2870 (C-H), 1672 (C=O) cm^{-1}.

2-[Benzyl(methyl)amino]anthracene-1,4-dione (4b). Yield 62%. Orange crystalline solid, mp 159–162 °C. Anal. calc. for $C_{22}H_{17}NO_2$ (327.38): C, 80.71, H, 5.23, N, 4.28. Found C, 80.93, H, 5.41, N, 4.37. ^{1}H NMR ($CDCl_3$) δ: 3.08 (3H, s, CH_3), 4.93 (2H, s, CH_2), 6.08 (1H, s, CH), 7.28-7.38 (5H, m, 5CH), 7.61-7.65 (2H, m, 2CH), 7.98-8.04 (2H, m, 2CH), 8.52 (1H, s, CH), 8.55 (1H, s, CH). ^{13}C NMR ($CDCl_3$) δ: 40.3 (CH_3), 57.6 (CH_2), 110.4, 127.0, 127.3, 127.7, 128.7, 128.9, 129.2, 129.3, 129.9 and 130.1 (10 CH), 127.6, 129.8, 134.4, 135.4 and 137.0 (five sp^2 tertiary C), 153.6 (C-N), 182.9 and 183.1 (2C=O). MS (EI, 70 eV): *m/z* (%)=327 (M^+, 100), 310 (30), 236 (57), 126 (23), 91 (50), 60 (39), 49 (22), 43 (47). HRESIMS: found *m/z* 328.1332; calc. for $C_{22}H_{17}NO_2$ $[M+H]^+$ 328.1338. IR (KBr): ν_{max}=3044 and 2913 (C-H), 1672 (C=O) cm^{-1}.

2-[Cyclohexyl(methyl)amino]anthracene-1,4-dione (4d). Yield 21%. Orange crystalline solid, mp 153–154 °C. Anal. calc. for $C_{21}H_{21}NO_2$ (319.40): C, 78.97, H, 6.63, N, 4.39. Found C, 79.13, H, 6.77, N, 4.52. ^{1}H NMR ($CDCl_3$) δ: 1.35-1.47 (2H, m, CH_2), 1.52-1.63 (2H, m, CH_2), 1.68-1.74 (2H, m, CH_2), 1.84-1.96 (4H, m, $2CH_2$), 2.97 (3H, s, CH_3), 4.10-4.18 (1H, m, CH), 6.03

(1H, s, CH), 7.59-7.66 (2H, m, 2CH), 7.98-8.04 (2H, m, 2CH), 8.52 (1H, s, CH), 8.54 (1H, s, CH). ^{13}C NMR ($CDCl_3$) δ: 25.6, 25.8 and 30.6 (three CH_2), 33.7 (CH_3), 60.5, 109.5, 126.7, 128.6, 129.1, 129.2, 129.9 and 130.0 (8 CH), 129.4, 130.3 134.3 and 135.3 (four sp^2 tertiary C), 154.7 (C-N), 182.8 and 183.6 (two C=O). MS (EI, 70 eV): *m*/*z* (%)=319 (M^+, 84), 276 (76), 236 (26), 196 (28), 165 (22), 155 (34), 152 (36), 139 (33), 127 (44), 111 (100), 101 (30), 97 (26), 83 (45), 70 (49), 55 (72). HRESIMS: found *m/z* 320.1651; calc. for $C_{21}H_{21}NO_2$ $[M+H]^+$ 320.1645. IR (KBr): ν_{max}=2920 and 2850 (C-H), 1679 (C=O) cm^{-1}.

2-[[2-(Dibenzylamino)ethyl](methyl)amino]anthracene-1,4-dione (4e). Yield 37%. Orange oil. Anal. calc. for $C_{31}H_{28}N_2O_2$ (460.57): C, 80.84, H, 6.13, N, 6.08. Found C, 80.73, H, 6.01, N, 6.00. ^{1}H NMR ($CDCl_3$) δ: 2.71 (2H, t, CH_2 *J* 5.9), 2.83 (3H, s, CH_3), 3.61 (4H, s, $2CH_2$), 3.93 (2H, t, CH_2 *J* 5.9), 5.84 (1H, s, CH), 7.08-7.35 (10H, m, 10CH), 7.58-7.67 (2H, m, 2CH), 7.95 (H, d, CH *J* 7.3), 8.03 (1H, d, CH *J* 8.1), 8.37 (1H, s, CH), 8.56 (1H, s, CH). ^{13}C NMR ($CDCl_3$) δ: 41.1 (one CH_3), 51.7, 52.2 and 59.0 (three CH_2), 109.2, 126.6, 127.1, 128.3, 128.5, 128.9, 129.1, 129.2, 129.8 and 130.0 (10 CH), 129.4, 130.1, 134.3, 135.3 and 139.0 (five sp^2 tertiary C), 153.4 (C-N), 182.6 and 183.3 (two C=O). MS (EI, 70 eV): *m*/*z* (%)=460 (M^+, 2), 237 (10), 210 (100), 181 (12), 152 (13), 106 (34), 91 (79), 65 (30), 43 (21). IR (KBr): ν_{max}=3027 and 2920 (C-H), 1673 (C=O) cm^{-1}.

2-[Isopropyl(methyl)amino]anthracene-1,4-dione (4f). Yield 53%. Orange crystalline solid, mp 106–109 °C. Anal. calc. for $C_{18}H_{17}NO_2$ (279.13): C, 77.40, H, 6.13, N, 5.01. Found C, 77.34, H, 5.89, N, 5.11. ^{1}H NMR ($CDCl_3$) δ: 1.28 (6H, d, $2CH_3$ *J* 6.6), 2.92 (3H, s, CH_3), 4.60-4.69 (1H, m, CH), 6.02 (1H, s, CH), 7.60-7.64 (2H, m, 2CH), 7.98-8.02 (2H, m, 2CH), 8.49 (1H, s, CH), 8.52 (1H, s, CH). ^{13}C NMR ($CDCl_3$) δ: 20.1 and 32.1 (two CH_3), 51.6, 109.7, 126.7, 128.5, 129.0, 129.3, 129.9 and 130.1 (8 CH), 129.6, 130.2, 134.3 and 135.3 (four sp^2 tertiary C), 154.6

(C-N), 182.8 and 183.4 (two C=O). MS (EI, 70 eV): *m/z* (%)=279 (M^+, 100), 264 (29), 43 (21). IR (KBr): ν_{max}=2965 and 2929 (C-H), 1671 (C=O) cm^{-1}.

3-Methyl-2-propyl-2,3-dihydronaphtho[2,3-*d*][1,3]thiazole-4,9-dione (1a). Yield 78%. Blue crystalline solid, mp 71–74 °C. Anal. calc. for $C_{15}H_{15}NO_2S$ (273.35): C, 65.91, H, 5.53, N, 5.12, S, 11.73. Found C, 66.12, H, 5.62, N, 5.33, S, 11.93. ^1H NMR ($CDCl_3$) δ: 0.98 (3H, t, CH_3 *J* 7.3), 1.46-1.74 (2H, m, CH_2), 1.84-1.93 (2H, m, CH_2), 3.40 (3H, s, CH_3), 5.14 (1H, t, CH *J* 5.5), 7.56-7.66 (2H, m, 2CH), 7.95-8.00 (2H, m, 2CH). ^{13}C NMR ($CDCl_3$) δ: 13.8 and 38.3 (two CH_3), 17.4 and 35.8 (two CH_2), 74.9, 125.1, 126.3, 132.1 and 133.7 (5 CH), 121.3 (C-S), 132.0 and 133.1 (two sp^2 tertiary C), 144.6 (C-N), 177.4 and 177.7 (two C=O). MS (EI, 70 eV): *m/z* (%)=273 (M^+, 13), 230 (100), 104 (29), 84 (35), 76 (51), 55 (64), 41(98). HRESIMS: found *m/z* 274.0896; calc. for $C_{15}H_{15}NO_2S$ $[M+H]^+$ 274.0901. IR (KBr): ν_{max}=2928 and 2874 (C-H), 1669 (C=O) cm^{-1}.

3-Methyl-2-phenyl-2,3-dihydronaphtho[2,3-*d*][1,3]thiazole-4,9-dione (1b). Yield 98%. Blue crystalline solid, mp 63–65 °C. Anal. calc. for $C_{18}H_{13}NO_2S$ (307.37): C, 70.34, H, 4.26, N, 4.56, 10.43. Found C, 70.12, H, 4.19, N, 4.70, S, 10.46. ^1H NMR ($CDCl_3$) δ: 3.24 (3H, s, CH_3), 6.10 (H, s, CH), 7.39-7.45 (3H, m, 3CH), 7.47-7.53 (2H, m, 2CH), 7.58-7.67 (2H, m, 2CH), 7.97-8.01 (2H, m, 2CH). ^{13}C NMR ($CDCl_3$) δ: 35.5 (one CH_3), 76.2, 125.1, 126.3, 127.5, 129.2, 129.7, 132.2 and 133.7 (8 CH), 121.5 (C-S), 132.1, 132.9 and 139.5 (three sp^2 tertiary C), 143.8 (C-N), 177.5 and 177.6 (two C=O). MS (EI, 70 eV): *m/z* (%)=307 (M^+, 30), 291 (17), 262 (14), 230 (100), 183 (32), 121 (28), 104 (54), 91 (47), 84 (44), 76 (84), 51 (65), 42 (41). HRESIMS: found *m/z* 308.0740; calc. for $C_{18}H_{13}NO_2S$ $[M+H]^+$ 308.0745. IR (KBr): ν_{max}=3026 and 2919 (C-H), 1654 (C=O) cm^{-1}.

3-Methyl-2,3-dihydronaphtho[2,3-*d*][1,3]thiazole-4,9-dione (1c). Yield 52%. Blue crystalline solid, mp 130–134 °C. Anal. calc. for $C_{12}H_9NO_2S$ (231.27): C, 62.32, H, 3.92, N, 6.06, S 13.86. Found C, 62.55, H, 3.99, N, 5.87, S 13.79. ^{1}H NMR ($CDCl_3$) δ: 3.37 (3H, s, CH_3), 4.92 (2H, s, CH_2), 7.52-7.64 (2H, m, 2CH), 7.89-7.95 (2H, m, 2CH). ^{13}C NMR ($CDCl_3$) δ: 36.8 (CH_3), 59.7 (CH_2), 123.0 (C-S), 125.1, 126.3, 132.2 and 133.7 (four CH), 131.9 and 133.0 (two sp^2 tertiary C), 145.4 (C-N), 177.4 and 177.5 (two C=O). MS (EI, 70 eV): *m*/*z* (%)=231 (M^+, 100), 216 (17), 187 (14), 140 (27), 92 (52), 76 (32). HRESIMS: found *m/z* 230.0270; calc. for $C_{12}H_9NO_2S$ [M-H]$^+$ 230.0776. IR (KBr): ν_{max}=2922 and 2855 (C-H), 1660 (C=O) cm^{-1}.

3'-Methyl-3'*H*-spiro[cyclohexane-1,2'-naphtho[2,3-*d*][1,3]thiazole]-4',9'-dione (1d). Yield 65%. Blue crystalline solid, mp 162–164 °C. Anal. calc. for $C_{17}H_{17}NO_2S$ (299.39): C, 68.20, H, 5.72, N, 4.68, S, 10.71. Found C, 68.50, H, 5.57, N, 4.53, S, 10.41. ^{1}H NMR ($CDCl_3$) δ: 1.56-1.73 (4H, m, $2CH_2$), 1.76-1.85 (2H, m, CH_2), 1.93-2.00 (2H, m, CH_2), 2.07-2.15 (2H, m, CH_2), 3.38 (3H, s, CH_3), 7.51-7.63 (2H, m, 2CH); 7.91-7.96 (2H, m, 2CH). ^{13}C NMR ($CDCl_3$) δ: 23.1, 23.2, 24.7, 36.5 and 36.6 (five CH_2), 30.8 (CH_3), 85.7, 132.2 and 132.9 (three sp^2 tertiary C), 119.2 (C-S), 124.9, 126.2, 131.9 and 133.6 (4 CH), 144.1 (C-N), 177.3 and 178.0 (two C=O). MS (EI, 70 eV): *m*/*z* (%)=299 (M^+, 31), 256 (100), 149 (18), 101 (23), 75 (18), 68 (23), 55 (26). HRESIMS: found *m/z* 300.1033; calc. for $C_{17}H_{17}NO_2S$ [M+Na]$^+$ 300.1058. IR (KBr): ν_{max}=2936 and 2855 (C-H), 1663 (C=O) cm^{-1}.

2-[2-(Dibenzylamino)methyl]-3-methyl-2,3-dihydronaphtho[2,3-*d*][1,3]thiazole-4,9-dione (1e). Yield 46%. Blue crystalline solid, mp 138–140 °C. Anal. calc. for $C_{27}H_{24}N_2O_2S$ (440.56): C, 73.61, H, 5.49, N, 6.36, S, 7.28. Found C, 73.47, H, 5.41, N, 6.17, S, 7.62. ^{1}H NMR ($CDCl_3$) δ: 2.83-2.90 (1H, m, CH_2-CH), 3.06-3.12 (1H, m, CH_2-CH), 3.26 (3H, s, CH_3), 3.70 (4H, dd, $2CH_2$ *J* 13.9, *J* 22.0), 4.86-4.91 (1H, m, CH), 7.25-7.39 (10H, m, 10CH), 7.51-7.62 (2H, m,

2CH), 7.89-7.95 (2H, m, 2CH). ^{13}C NMR ($CDCl_3$) δ: 37.2 (CH_3), 60.0 and 60.4 (two CH_2), 74.1, 125.0, 126.2, 127.5, 128.5, 129.1, 132.1 and 133.6 (8 CH), 122.3 (C-S), 132.0, 133.0 and 138.7 (three sp^2 tertiary C), 144.6 (C-N), 177.4 and 177.7 (two C=O). MS (EI, 70 eV): *m*/*z* (%)=441 (10), 440 (M^+, 3), 439 (23), 347 (60), 231 (52), 210 (100), 196 (35), 132 (33), 106 (87), 91 (78), 65 (37), 51 (30). HRESIMS: found *m/z* 441.1631; calc. for $C_{27}H_{24}N_2O_2S$ $[M+H]^+$ 441.1637. IR (KBr): ν_{max}=3029 and 2934 (C-H), 1665 (C=O) cm^{-1}.

2,2,3-Trimethyl-2,3-dihydronaphtho[2,3-*d*][1,3]thiazole-4,9-dione (1f). Yield 72%. Blue crystalline solid, mp 164–166 °C. Anal. calc. for $C_{14}H_{13}NO_2S$ (259.32): C, 64.84, H, 5.05, N, 5.40, S, 12.36. Found C, 65.06, H, 5.18, N, 5.21, S, 11.96. ^{1}H NMR ($CDCl_3$) δ: 1.76 (6H, s, 2CH_3), 3.38 (3H, s, CH_3), 7.54-7.65 (2H, m, 2CH), 7.94-7.98 (2H, m, 2CH). ^{13}C NMR ($CDCl_3$) δ: 29.0 and 30.3 (two CH_3), 78.1, 132.2 and 132.9 (three sp^2 tertiary C), 119.7 (C-S), 124.9, 126.2, 132.0 and 133.6 (four CH), 143.2 (C-N), 177.5 and 177.8 (two C=O). MS (EI, 70 eV): *m*/*z* (%)=259 (M^+, 18), 244 (100), 186 (10), 75 (10), 56 (42), 43 (10). HRESIMS: found *m/z* 259.0662; calc. for $C_{14}H_{13}NO_2S$ $[M]^+$ 259.0667. IR (KBr): ν_{max}=2976 and 2927 (C-H), 1665 (C=O) cm^{-1}.

3-Methyl-2-propyl-2,3-dihydroanthra[2,3-*d*][1,3]thiazole-4,11-dione (2a). Yield 67%. Blue crystalline solid, mp 140–142 °C. Anal. calc. for $C_{19}H_{17}NO_2S$ (323.41): C, 70.56, H, 5.30, N, 4.33, S, 9.91. Found C, 70.42, H, 5.12, N, 4.53, S, 9.89. ^{1}H NMR ($CDCl_3$) δ: 1.00 (3H, t, CH_3 *J* 7.3), 1.48-1.55 (2H, m, CH_2), 1.88-1.95 (2H, m, CH_2), 3.47 (3H, s, CH_3), 5.18 (H, t, CH *J* 5.9), 7.57-7.65 (2H, m, 2CH), 7.95-7.99 (2H, m, 2CH), 8.43 (1H, s, CH), 8.44 (1H, s, CH). ^{13}C NMR ($CDCl_3$) δ: 13.8 and 38.3 (two CH_3), 17.4 and 35.8 (two CH_2), 74.9, 126.4, 128.7, 128.8, 129.4, 129.9 and 130.1 (7 CH), 129.2, 129.3, 134.2 and 135.1 (four sp^2 tertiary C), 124.1 (C-S), 145.7 (C-N), 176.8 and 177.2 (two C=O). MS (EI, 70 eV): *m*/*z* (%)=323 (M^+, 27), 306 (11), 281 (100),

140 (8), 126 (18), 101 (13). HRESIMS: found *m/z* 324.1053; calc. for $C_{19}H_{17}NO_2S$ $[M+H]^+$ 324.1058. IR (KBr): ν_{max}=2960 and 2873 (C-H), 1658 (C=O) cm^{-1}.

3-Methyl-2-phenyl-2,3-dihydroanthra[2,3-*d*][1,3]thiazole-4,11-dione (2b). Yield 68%. Yellow crystalline solid, mp 78–80 °C. Anal. calc. for $C_{22}H_{15}NO_2S$ (357.43): C, 73.93, H, 4.23, N, 3.92, S, 8.97. Found C, 73.65, H, 4.03, N, 3.91, S, 9.02. ^{1}H NMR ($CDCl_3$) δ: 3.29 (3H, s, CH_3), 6.12 (H, s, CH), 7.40-7.46 (3H, m, 3CH), 7.50-7.54 (2H, m, 2CH), 7.60-7.64 (2H, m, 2CH), 7.95-7.99 (2H, m, 2CH), 8.44 (1H, s, CH), 8.45 (1H, s, CH). ^{13}C NMR ($CDCl_3$) δ: 35.5 (CH_3), 76.2, 126.4, 127.5, 128.7, 128.8, 129.2, 129.4, 129.7, 129.9 and 130.0 (10 CH), 124.2 (C-S), 129.3, 129.8, 134.2, 135.0 and 139.4 (five sp^2 tertiary C), 145.0 (C-N), 176.6 and 177.0 (two C=O). MS (EI, 70 eV): *m*/*z* (%)=357 (M^+, 83), 280 (100), 126 (25), 57 (17), 43 (37). HRESIMS: found *m/z* 356.0740; calc. for $C_{22}H_{15}NO_2S$ $[M-H]^+$ 356.0745. IR (KBr): ν_{max}=3032 and 2924 (C-H), 1652 (C=O) cm^{-1}.

3-Methyl-2,3-dihydroanthra[2,3-*d*][1,3]thiazole-4,11-dione (2c). Yield 52%. Blue crystalline solid, mp 247–248 °C. Anal. calc. for $C_{16}H_{11}NO_2S$ (281.33): C, 68.31, H, 3.94, N, 4.98, S, 11.40. Found C, 68.56, H, 4.09, N, 5.06, S, 11.22. ^{1}H NMR ($CDCl_3$) δ: 3.41 (3H, s, CH_3), 4.94 (2H, s, CH_2), 7.55-7.63 (2H, m, 2CH), 7.88-7.96 (2H, m, 2CH), 8.32 (1H, s, CH), 8.35 (1H, s, CH). ^{13}C NMR ($CDCl_3$) δ: 36.9 (CH_3), 59.8 (CH_2), 125.7 (C-S), 126.4, 128.7, 128.8, 129.5, 129.9 and 130.1 (6 CH), 129.2, 129.3, 134.1 and 135.0 (four sp^2 tertiary C), 146.5 (C-N), 176.6 and 176.9 (two C=O). MS (EI, 70 eV): *m*/*z* (%)=281 (M^+, 100), 182 (10), 155 (10), 140 (12), 126 (15), 43 (40). HRESIMS: found *m/z* 280.0427; calc. for $C_{16}H_{11}NO_2S$ $[M-H]^+$ 280.0432. IR (KBr): ν_{max}=2923 and 2852 (C-H), 1657 (C=O) cm^{-1}.

3-Methyl-3*H*-spiro[anthra[2,3-*d*][1,3]thiazole-2,1'-cyclohexane]-4,11-dione (2d). Yield 95%. Blue crystalline solid, mp 202–204 °C. Anal. calc. for $C_{21}H_{19}NO_2S$ (349.45): C, 72.18, H, 5.48, N, 4.01, S, 9.18. Found C, 72.33, H, 5.66, N, 4.13, S, 8.63. ^1H NMR ($CDCl_3$) δ: 1.62-1.76 (4H, m, $2CH_2$), 1.80-1.88 (2H, m, CH_2), 1.96-2.04 (2H, m, CH_2), 2.12-2.19 (2H, m, CH_2), 3.46 (3H, s, CH_3), 7.56-7.64 (2H, m, 2CH), 7.93-7.99 (2H, m, 2CH), 8.42 (1H, s, CH), 8.43 (1H, s, CH). ^{13}C NMR ($CDCl_3$) δ: 23.0, 23.1, 24.7, 36.5 and 36.6 (five CH_2), 30.9 (CH_3), 85.8, 129.3, 129.8, 134.2 and 135.1 (five sp^2 tertiary C), 122.1 (C-S); 126.2, 128.6, 128.8, 129.4, 129.9 and 130.1 (6 CH); 145.4 (C-N), 177.0 and 177.2 (two C=O). MS (EI, 70 eV): *m*/*z* (%)=349 (M^+, 51), 306 (100), 281 (100), 177 (30), 146 (19), 127 (22), 68 (21), 55 (37), 40 (47). HRESIMS: found *m/z* 350.1209; calc. for $C_{21}H_{19}NO_2S$ $[M+H]^+$ 350.1215. IR (KBr): ν_{max}=2932 and 2856 (C-H), 1664 (C=O) cm^{-1}.

2-[2-(Dibenzylamino)methyl]-3-methyl-2,3-dihydroanthra[2,3-*d*][1,3]thiazole-4,11-dione (2e). Yield 60%. Blue crystalline solid, mp 157–161 °C. Anal. calc. for $C_{31}H_{26}N_2O_2S$ (490.62): C, 75.89, H, 5.34, N, 5.71, S, 6.54. Found C, 76.08, H, 5.56, N, 5.89, S, 6.38. ^1H NMR ($CDCl_3$) δ: 2.87-2.94 (1H, m, CH_2-CH), 3.09-3.16 (1H, m, CH_2-CH), 3.32 (3H, s, CH_3), 3.72 (4H, dd, $2CH_2$ J 13.7, J 21.1), 4.90-4.94 (1H, m, CH), 7.24-7.41 (10H, m, 10CH), 7.56-7.63 (2H, m, 2CH), 7.92-7.96 (2H, m, 2CH), 8.38 (1H, s, CH), 8.39 (1H, s, CH). ^{13}C NMR ($CDCl_3$) δ: 37.2 (one CH_3), 60.0 and 60.5 (two CH_2), 74.2, 126.4, 127.5, 128.6, 128.7, 128.8, 128.9, 129.1, 130.0 and 130.1 (10 CH), 125.1 (C-S), 129.3, 129.4, 134.2, 135.0 and 138.6 (five sp^2 tertiary C), 146.0 (C-N), 176.6 and 177.3 (two C=O). MS (EI, 70 eV): *m*/*z* (%)=490 (M^+, 17), 397 (9), 280 (55), 210 (60), 196 (12), 155 (10), 108 (43), 91 (100), 67 (27), 43 (15). HRESIMS: found *m/z* 491.1788; calc. for $C_{31}H_{26}N_2O_2S$ $[M+H]^+$ 491.1793. IR (KBr): ν_{max}=3031 and 2920 (C-H), 1661 (C=O) cm^{-1}.

2,2,3-Trimethyl-2,3-dihydroanthra[2,3-*d*][1,3]thiazole-4,11-dione (2f). Yield 68%. Blue crystalline solid, mp 202–205 °C. Anal. calc. for $C_{18}H_{15}NO_2S$ (309.38): C, 69.88, H, 4.89, N, 4.53, S, 10.36. Found C, 70.12, H, 5.06, N, 4.31, S, 9.96. ^{1}H NMR ($CDCl_3$) δ: 1.77 (6H, s, $2CH_3$), 3.43 (3H, s, CH_3), 7.56-7.63 (2H, m, 2CH), 7.91-7.99 (2H, m, 2CH), 8.40 (1H, s, CH), 8.42 (1H, s, CH). ^{13}C NMR ($CDCl_3$) δ: 29.2 and 30.4 (two CH_3), 78.2, 129.2, 129.6, 134.1 and 135.0 (five sp^2 tertiary C), 122.5 (C-S), 126.2, 128.6, 128.7, 129.4, 129.9 and 130.0 (6 CH), 144.4 (C-N), 177.0 and 177.1 (two C=O). MS (EI, 70 eV): *m/z* (%)=309 (M^+, 20), 295 (100), 126 (37), 85 (20), 44 (13). HRESIMS: found *m/z* 310.0896; calc. for $C_{18}H_{16}NO_2S$ $[M+H]^+$ 310.0902. IR (KBr): ν_{max}=2964 and 2923 (C-H), 1653 (C=O) cm^{-1}.

2-[Butyl(methyl)amino]-3-chloronaphthoquinone (7a). Yield 98%. Red crystalline solid, mp 92–94 °C. Anal. calc. for $C_{15}H_{16}ClNO_2$ (277.75): C 64.87; H 5.81; Cl 12.76; N 5.04. Found C, 73.23, H, 4.67, Cl, 9.62, N, 4.03. ^{1}H NMR ($CDCl_3$) δ: 0.92 (3H, t, CH_3 *J* 7.3), 1.26-1.40 (2H, m, CH_2), 1.61-1.71 (2H, m, CH_2), 3.19 (3H, s, CH_3), 3.57 (2H, t, CH_2 *J* 7.3), 7.63-7.72 (2H, m, 2CH), 8.00 (H, d, CH *J 6.6*), 8.11 (H, d, CH *J* 7.3). ^{13}C NMR ($CDCl_3$) δ: 13.9 and 42.2 (two CH_3), 19.9, 30.6 and 55.4 (three CH_2), 122.0 (C-Cl), 126.5, 126.9, 132.9 and 134.0 (four CH), 131.7 and 131.8 (two sp^2 tertiary C), 151.6 (C-N), 178.1 and 182.3 (two C=O). MS (EI, 70 eV): *m/z* (%)=279 (M+2, 33), 277 (M^+, 81), 234 (100), 222 (30), 186 (22), 135 (29), 55 (35). IR (KBr): ν_{max}=2948 and 2869 (C-H), 1678 (C=O) cm^{-1}.

2-[Butyl(methyl)amino]-3-chloroanthracene-1,4-dione (9a). Yield 61%. Red crystalline solid, mp 185–187 °C. Anal. calc. for $C_{19}H_{18}ClNO_2$ (327.80): C, 69.62, H, 5.53, Cl, 10.82, N, 4.27. Found C, 69.43, H, 5.33, Cl, 10.98, N, 4.44. ^{1}H NMR ($CDCl_3$) δ: 0.94 (3H, t, CH_3 *J* 7.3), 1.28-1.42 (2H, m, CH_2), 1.64-1.74 (2H, m, CH_2), 3.23 (3H, s, CH_3), 3.61 (2H, t, CH_2 *J* 7.3), 7.62-7.69 (2H, m, 2CH), 7.99-8.05 (2H, m, 2CH), 8.53 (1H, s, CH), 8.62 (1H, s, CH). ^{13}C NMR ($CDCl_3$) δ:

13.9 and 42.3 (two CH_3), 20.0, 30.7 and 55.5 (three CH_2), 124.0 (C-Cl), 128.0, 128.5, 134.6 and 135.2 (four sp^2 tertiary C), 128.3, 129.1, 129.4, 129.6 and 130.0 (two signals) (6 CH), 152.9 (C-N), 178.0 and 182.0 (two C=O). MS (EI, 70 eV): *m/z* (%)=329 (M+2, 10), 327 (M^+, 27), 309 (10), 284 (34), 149 (17), 91 (67), 86 (87), 83 (100), 76 (84), 63 (44), 53 (32). IR (KBr): ν_{max}=2953 and 2868 (C-H), 1674 (C=O) cm^{-1}.

3-Butyl-2-thioxo-2,3-dihydroanthra[2,3-*d*][1,3]thiazole-4,11-dione (10a). Yield 36%. Red crystalline solid, mp 183–185 °C. Anal. calc. for $C_{19}H_{15}NO_2S_2$ (353.46): C, 64.56, H, 4.28, N, 3.96, S, 18.14. Found C, 64.75, H, 4.37, N, 4.04, S, 17.67. ^1H NMR ($CDCl_3$) δ: 1.04 (3H, t, CH_3 *J* 7.3), 1.49-1.59 (2H, m, CH_2), 1.75-1.85 (2H, m, CH_2), 4.81 (2H, t, CH_2 *J* 8.1), 7.73-7.76 (2H, m, 2CH), 8.07-8.10 (2H, m, 2CH), 8.65 (1H, s, CH), 8.72 (1H, s, CH). ^{13}C NMR ($CDCl_3$) δ: 13.6 (CH_3), 19.9, 29.9 and 48.5 (three CH_2), 118.6 (C-S), 127.2, 128.3, 134.6 and 134.7 (four sp^2 tertiary C), 129.1, 130.1, 130.2, 130.3, 130.4 and 130.7 (6 CH), 141.6 (C-N), 173.3 and 175.2 (two C=O), 189.8 (C=S). MS (EI, 70 eV): *m/z* (%)=353 (M^+, 31), 320 (66), 297 (100), 239 (15), 155 (13), 126 (32). HRESIMS: found *m/z* 354.0637; calc. for $C_{19}H_{15}NO_2S_2$ $[M+H]^+$ 354.0622. IR (KBr): ν_{max}=2955 and 2871 (C-H), 1667 (C=O) cm^{-1}.

2-Chloro-3-(dimethylamino)anthracene-1,4-dione (9c). Yield 59%. Red crystalline solid, mp 185–187 °C. Anal. calc. for $C_{16}H_{12}ClNO_2$ (285.72): C, 67.27, H, 4.23, Cl, 12.41, N, 4.90. Found C, 67.46, H, 4.40, Cl, 12.22, N, 5.15. ^1H NMR ($CDCl_3$) δ: 3.30 (6H, s, $2CH_3$), 7.64-7.67 (2H, m, 2CH), 8.01-8.05 (2H, m, 2CH), 8.54 (1H, s, CH), 8.63 (1H, s, CH). ^{13}C NMR ($CDCl_3$) δ: 44.4 (CH_3), 122.0 (C-Cl), 127.7, 128.8, 134.4 and 135.0 (four sp^2 tertiary C), 128.0, 128.9, 129.2, 129.4, 129.8 and 129.9 (6 CH), 152.0 (C-N), 177.6 (C=O), 181.6 (C=O). MS (EI, 70 eV): *m/z* (%)=287 (M+2, 16), 285 (M^+, 38), 270 (19), 250 (17), 179 (18), 167 (20), 149 (100), 126 (20), 112 (20), 92 (61), 82 (30). IR (KBr): ν_{max}=2925 (C-H), 1674 (C=O) cm^{-1}.

3-Methyl-2-thioxo-2,3-dihydroanthra[2,3-*d*][1,3]thiazole-4,11-dione (10c). Yield 14%. Red crystalline solid, mp 314–317 °C. Anal. calc. for $C_{16}H_9NO_2S_2$ (311.38): C, 61.72, H, 2.91, N, 4.50. Found C, 61.85, H, 3.12, N, 4.68. ^{1}H NMR ($CDCl_3$) δ: 4.22 (3H, s, CH_3), 7.74-7.77 (2H, m, 2CH), 8.08-8.12 (2H, m, 2CH), 8.68 (1H, s, CH), 8.75 (1H, s, CH). MS (EI, 70 eV): *m/z* (%)=311 (M^+, 100), 207 (20), 179 (28), 152 (21), 126 (80), 76 (26). HRESIMS: found *m/z* 312.0168; calc. for $C_{16}H_9NO_2S_2$ $[M+H]^+$ 312.0153. IR (KBr): ν_{max}=2925 and 2853 (C-H), 1665 (C=O) cm^{-1}.

2-Chloro-3-[cyclohexyl(methyl)amino]anthracene-1,4-dione (9d). Yield 74%. Red crystalline solid, mp 164–167 °C. Anal. calc. for $C_{21}H_{20}ClNO_2$ (353.84): C, 71.28, H, 5.70, Cl, 10.02, N, 3.96. Found C, 71.37, H, 5.85, Cl, 9.82, N, 4.11. ^{1}H NMR ($CDCl_3$) δ: 1.32-1.44 (2H, m, CH_2), 1.68-1.72 (4H, m, 2CH_2); 1.86-1.90 (2H, m, CH_2); 1.99-2.03 (2H, m, CH_2); 3.10 (3H, s, CH_3); 3.69-3.75 (H, m, CH); 7.64-7.68 (2H, m, 2CH), 8.00-8.06 (2H, m, 2CH), 8.56 (1H, s, CH), 8.64 (1H, s, CH). ^{13}C NMR ($CDCl_3$) δ: 25.7, 26.1 and 31.4 (three CH_2), 35.9 (CH_3), 62.9, 128.3, 129.1, 129.4, 129.5 and 130.0 (two signals) (7 CH), 125.5 (C-Cl), 128.0, 128.5, 134.6 and 135.2 (four sp^2 tertiary C), 153.6 (C-N), 178.0 and 182.1 (two C=O). MS (EI, 70 eV): *m/z* (%)=355 (M+2, 16), 353 (M^+, 40), 319 (14), 274 (14), 149 (10), 64 (48), 58 (84), 43 (100). IR (KBr): ν_{max}=2927 and 2852 (C-H), 1671 (C=O) cm^{-1}.

3-Cyclohexyl-2-thioxo-2,3-dihydroanthra[2,3-*d*][1,3]thiazole-4,11-dione (10d). Yield 9%. Red crystalline solid, mp 275–278 °C. Anal. calc. for $C_{21}H_{17}NO_2S_2$ (379.50): C, 66.46, H, 4.52, N, 3.69, S, 16.90. Found C, 66.19, H, 4.68, N, 3.66, S, 16.67. ^{1}H NMR ($CDCl_3$) δ: ^{1}H NMR ($CDCl_3$) δ: 1.43-1.50 (2H, m, CH_2), 1.77-1.85 (3H, m, 2CH_2), 1.93-2.01 (4H, m, 2CH_2), 2.50-2.70 (1H, m, 2CH_2), 5.67-5.75 (H, m, CH), 7.72-7.76 (2H, m, 2CH), 8.06-8.10 (2H, m, 2CH),

8.63 (1H, s, CH), 8.72 (1H, s, CH). ^{13}C NMR ($CDCl_3$) δ: 24.9, 26.3 and 28.0 (three CH_2), 62.3, 129.0, 130.2, 130.3, 130.4, 130.5, and 131.1 (7 CH), 127.4, 129.2, 134.7 and 135.1 (four sp^2 tertiary C), 130.6 (C-S), 137.3 (C-N), 175.7 (C=O), 190.9 (C=S). MS (EI, 70 eV): *m/z* (%)=379 (M^+, 20), 298 (100), 154 (8), 139 (8), 126 (7), 67 (12), 55 (12), 43 (29). HRESIMS: found *m/z* 380.0773; calc. for $C_{21}H_{17}NO_2S_2$ $[M+H]^+$ 380.0779. IR (KBr): ν_{max}=2932 and 2848 (C-H), 1668 (C=O) cm^{-1}.

3-Methyl-2-thioxo-2,3-dihydronaphtho[2,3-*d*][1,3]thiazole-4,9-dione (11). Yield 74%. Red crystalline solid, mp 196–198 °C. Anal. calc. for $C_{12}H_7NO_2S_2$ (261.32): C, 55.15, H, 2.70, N, 5.36, S, 24.54. Found C, 55.07, H, 2.76, N, 5.22, S, 24.62. ^{1}H NMR ($CDCl_3$) δ: 4.15 (3H, s, CH_3), 7.78-7.85 (2H, m, 2CH), 8.14-8.17 (1H, m, CH), 8.19-8.22 (1H, m, CH). ^{13}C NMR ($CDCl_3$) δ: 36.3 (CH_3), 126.8, 127.8, 134.5 and 134.7 (4 CH), 131.5 and 132.0 (two sp^2 tertiary C), 133.2 (C-S), 141.0 (C-N), 174.4 and 175.8 (two C=O), 190.4 (C=S). MS (EI, 70 eV): *m/z* (%)=261 (M^+, 29), 157 (21), 129 (35), 104 (35), 76 (100), 50 (53). HRESIMS: found *m/z* 291.9992; calc. for $C_{12}H_7NO_2S_2$ $[M+H]^+$ 291.9996. IR (KBr): ν_{max}=2926 and 2854 (C-H), 1671 (C=O) cm^{-1}.

3-Methyl-2-thioxo-2,3-dihydroanthra[2,3-*d*][1,3]thiazole-4,11-dione (10c). Yield 67%.

5-Methyl-2,3,4,5-tetrahydro-1*H*-benzo[*b*]carbazole-6,11-dione (15). Yield 78%. Yellow crystalline solid, mp 182–184 °C, lit. [16] mp 183–184 °C.

5-Methyl-2,3,4,5-tetrahydro-1*H*-naphtho[2,3-*b*]carbazole-6,13-dione (16). Yield 78%. Yellow crystalline solid, mp 253–255 °C. Anal. calc. for $C_{21}H_{17}NO_2$ (315.37): C, 79.98, H, 5.43, N, 4.44. Found C, 79.82, H, 5.58, N, 4.46. ^{1}H NMR ($CDCl_3$) δ: 1.82-1.83 (2H, m, CH_2), 1.90-

1.92 (2H, m, CH_2), 2.59-2.63 (2H, m, CH_2), 2.93-2.97 (2H, m, CH_2), 3.99 (3H, s, CH_3), 7.60-7.64 (2H, m, 2CH), 8.00-8.04 (2H, m, 2CH), 8.62 (1H, s, CH), 8.63 (1H, s, CH). ^{13}C NMR ($CDCl_3$) δ: 22.0, 22.4, 29.8 and 32.5 (four CH_2), 22.8 (CH_3), 121.5, 126.2, 131.1, 131.5, 134.7 and 134.8 (6 sp^2 tertiary C), 128.0, 128.1, 128.7 (two signals), 129.9 and 130.0 (6 CH), 140.2 and 158.2 (two C-N), 175.4 and 181.8 (two C=O). MS (EI, 70 eV): *m/z* (%)=315 (M^+, 83), 300 (35), 287 (28), 154 (20), 127 (35), 84 (100), 71 (71), 55 (71). HRESIMS: found *m/z* 316.1332; calc. for $C_{21}H_{17}NO_2$ $[M+H]^+$ 316.1338. IR (KBr): ν_{max}=2937 and 2851 (C-H), 1654 (C=O) cm^{-1}.
